# Transnasal humidified rapid insufflation ventilatory exchange vs. facemask oxygenation in elderly patients undergoing general anaesthesia: a randomized controlled trial

**DOI:** 10.1038/s41598-020-62716-2

**Published:** 2020-04-01

**Authors:** Zhen Hua, Zhen Liu, Yang Li, Hongye Zhang, Ming Yang, Mingzhang Zuo

**Affiliations:** 1 Department of Anaesthesiology, Beijing Hospital, National Center of Gerontology, Institute of Geriatric Medicine, Chinese Academy of Medical Science, Beijing, P.R. China; 2 Department of Anaesthesiology, Beijing Changping Hospital, Beijing, P.R. China

**Keywords:** Diseases, Health care, Medical research, Signs and symptoms

## Abstract

Transnasal humidified rapid insufflation ventilator exchange (THRIVE) may be effective in delaying hypoxia, but the efficacy of THRIVE for oxygenation in elderly patients under general anaesthesia has not been assessed. This study assessed whether THRIVE prolonged the apnoea time in the elderly patients after induction. This was a single centre, two-group, randomized controlled trial. 60 patients (65 to 80 years of age) with American Society of Anesthesiologists (ASA) grades I ~ III who required tracheal intubation or the application of a laryngeal mask under general anaesthesia were randomly allocated to receive oxygenation using THRIVE (100% oxygen, 30~70 litres min^−1^) or a facemask (100% oxygen, 10 litres min^−1^) during the pre-oxygenation period and during apnoea. The apnoea time, which was defined as the time from the cessation of spontaneous breathing until the SpO_2_ decreased to 90% or the apnoea time reached 10 minutes was recorded as the primary outcome. No significant differences were found on the baseline characteristics between the groups. The apnoea time was significantly increased (*P* < 0.01) in the THRIVE group. The median (interquartile range) apnoea times were 600 (600–600) s in the THRIVE group and 600 (231.5–600) s in the facemask group. No significant differences were found in the PaO_2_, PaCO_2_ and vital parameters between the THRIVE and facemask groups. No increased occurrence of complications, including haemodynamic instability, resistant arrhythmia or nasal discomfort, were reported in both the THRIVE group and the facemask group. THRIVE prolongs the apnoea time in elderly patients. THRIVE may be a more effective method for pre-oxygenation than a facemask in the elderly without pulmonary dysfunction.

## Introduction

Hypoxaemia is an emergency situation that may occur during the induction of anaesthesia and can lead to devastating consequences, such as arrhythmia, myocardial infarction and cerebral infarction, especially in elderly patients^1–3^. To prevent hypoxaemia, pre-oxygenation with 100% oxygen using a facemask is routinely performed before the induction of anaesthesia to extend the desaturation time^4,5^.

In elderly patients, physiological changes, including the loss of lung static recoil forces, the stiffening of the chest wall and diminished alveolar surface area, result in decreased oxygen stores^6^. A clinical history of coronary heart disease and stroke, which are not uncommon in the elderly, increase the incidence of severe complications caused by hypoxaemia^7–9^. Moreover, toothlessness decreases the efficiency of mask ventilation. Cervical osteoarthritis and snoring may extend the intubation time in elderly patients^5,10^. Therefore, obtaining sufficient and extended oxygenation is of greater importance for elderly patients than for younger patients.

Transnasal humidified rapid insufflation ventilator exchange (THRIVE) is defined as a technique that uses warmed and humidified oxygen administered via high-flow nasal cannula to achieve apnoeic oxygenation and ventilation^11^. An increased oxygen flow rate is associated with an increased fraction of inspired oxygen^12,13^. By using a nasal cannula, oxygen can be delivered at rates of 70 litres min^−1^ during THRIVE^14^. In contrast to the facemask, THRIVE can provide a continuous oxygen supply throughout the intubation procedure. Case reports and previous researches have suggested that THRIVE promoted oxygenation in children, obese patients and difficult airway management^10,15–17^. However, few studies focused on the oxygenation effect of THRIVE on the elderly patients. In this study, we hypothesized that THRIVE may have a better effect on oxygenation and increase the apnoea time in elderly patients during the induction of anaesthesia. We designed a randomized controlled study comparing pre-oxygenation using THRIVE with pre-oxygenation via a facemask.

The aim of this study was to investigate whether the apnoea time can be extended by THRIVE when THRIVE is used for pre-oxygenation and during the apnoea time rather than a facemask in elderly patients. The secondary aim was to investigate the effects of THRIVE on oxygenation and haemodynamic during pre-oxygenation and apnoea.

## Methods

All methods were carried out in accordance with relevant guidelines and regulations.

### Study design

This study was approved by the Institutional Review Board of Beijing Hospital, Beijing, China (approval number: 2017BJYYEC-063-03) on 28 September, 2017 and written informed consent was obtained from all subjects participating in the trial. The trial was registered at the Chinese Clinical Trial Registry (ChiCTR-IOR-17012954) on October 12, 2017. The trail protocol can be accessed on http://www.chictr.org.cn. The work has been reported in line with Consolidated Standards of Reporting Trials (CONSORT) Guidelines (supplementary materials).

This was a single centre, prospective, single-blinded, randomized controlled trial conducted between October 27, 2017 and July 10, 2018 in Beijing Hospital, Beijing, China.

### Patients selection

Eligible participants were elderly patients aged ≥65 and <80 years with ASA grade I ~ III who required tracheal intubation or the application of a laryngeal mask under general anaesthesia. The exclusion criteria were as follows: refusal to join the study, obesity (BMI > 35 kg m^−2^), a diagnosis of obstructive sleep apnoea syndrome, known or anticipated difficulty with the airway that necessitated intubation while awake, influence of acute pain on autonomous respiration, and undergoing thoracic surgery.

Participants were randomized to either the THRIVE group or the facemask group with balanced randomization (1:1). Randomization was accomplished using a scratch card with group allocation printed on it. There was a one-to-one correspondence between each patient and the scratch card. The scratch card remained unscratched until pre-oxygenation would be performed. The scratch card was provided by the statistics department of Peking University First Hospital.

### Interventions

Pre-intervention was performed in all patients by infusing dexmedetomidine (0.3 µg kg^−1^) intravenously in 15 minutes before pre-oxygenation. Radial artery puncture and catheterization were performed during the infusion of dexmedetomidine. The bispectral index (BIS) monitor was used in all patients. SpO_2,_ blood pressure, heart rate and arterial blood gases (ABG) were recorded as the baseline data after the dexmedetomidine infusion was finished. The examination of ABG included the result of hemoglobin. In this study, all patients remained in the supine position before surgery.

Patients in the THRIVE group were pre-oxygenated for 5 minutes using the high flow nasal cannula (Fisher & Paykel Healthcare, New Zealand). The flow rate was 100% oxygen at 30 litres min^−1^ during pre-oxygenation. Patients in the facemask group were pre-oxygenated for 5 minutes using a facemask at a flow rate of 100% oxygen at 10 litres min^−1^ without positive airway pressure. SpO_2,_ blood pressure, heart rate and ABG were recorded 3 minutes after pre-oxygenation.

The induction of anaesthesia was performed after pre-oxygenation. Specifically, 2~3 mg kg^−1^ propofol or 0.3 mg kg^−1^ etomidate, 0.2~0.4 µg kg^−1^ sufentanil and 0.1~0.2 mg kg^−1^ cisatracurium were administered intravenously in all patients included. The BIS values were maintained between 40 to 60 in all patients after induction. Patients in both groups received jaw support and placement of oropharyngeal airways to ensure an open airway when consciousness and spontaneous breathing ceased. In the THRIVE group, the flow rate increased to 70 litres min^−1^ 100% oxygen after the induction of anaesthesia and remained at 70 litres min^−1^. In the facemask group, the facemask was placed on the face tightly, and the flow rate remained at 10 litres min^−1^ 100% oxygen without positive airway pressure. Once the SpO_2_ reached 90% or the apnoeic time reached 10 minutes, mechanical ventilation (tidal volume: 8 ml kg;^−1^ respiratory rate: 12 min;^−1^ PEEP: 0 cm H_2_O) was performed in all patients. The high-flow nasal cannula in the THRIVE group was removed before mechanical ventilation. Patients in both groups received jaw support and placement of oropharyngeal airways during mechanical ventilation. When the SpO_2_ increased to 100%, patients underwent endotracheal intubation or placement of the laryngeal mask.

If the patient’s heart rate was <45 bpm, atropine 0.3~0.5 mg was injected intravenously. If the patient’s systolic blood pressure (SBP) decreased by more than 30% of the baseline value or decreased to less than 90 mmHg, 6 mg ephedrine was injected intravenously. If the patient’s SBP increased by more than 30% of the baseline value, urapidil 10 mg or isosorbide mononitrate 2 mg was injected intravenously. If resistant arrhythmia manifested during apnoea, the study would be stopped immediately and the patient would be ventilated and excluded from this study.

### Outcomes

The primary outcome was the apnoea time, which was defined as the time from the cessation of spontaneous breathing to the beginning of mechanical ventilation. To ensure the safety of patients involved in this study, mechanical ventilation would be performed when the apnoea time reached 10 minutes or the SpO_2_ decreased to 90% according to previous studies^4, 19^.ABG was recorded when the SpO_2_ decreased to 90% or the apnoea time reached 10 minutes before the application of mechanical ventilation and 3 minutes after pre-oxygenation. The time from the initiation of mechanical ventilation until the time when the SpO_2_ reached the baseline level was recorded and defined as the re-oxygenation time. Newly presenting arrhythmia during the induction process and nasal discomfort during the first 24 hours after the operation were recorded. ECG was performed in all patients before the elective operation. Newly presenting arrhythmia was defined as an arrhythmia that had not been diagnosed before pre-oxygenation. Nasal discomfort was defined as felling dry, pain or itch in the nasal cavity.

### Sample size and statistical analysis

The sample size was based on the apnoea time in adults reported in previously published data^4,14,18^. We calculated that 23 participants were required, assuming a type I error of 5%, a power >0.9, a difference of 2 minutes and a standard deviation of 2 minutes. Given an anticipated dropout rate of 20%, 30 patients were included in each group.

The Shapiro-Wilk assess was used to test for the normal distribution of the data. The apnoea time was analysed using Kaplan-Meier method. Data were analysed with unpaired Student’s t-tests, Chi-square test, Mann-Whitney U test or Fisher’s exact test, as appropriate. Data are presented as the mean (SD) or median (interquartile range). A two-sided *P-*value < 0.05 was considered statistically significant. The statistical analyses were performed with SPSS 24.0 (IBM, New York, USA).

## Results

### Clinical features of patients

**Figure 1:**
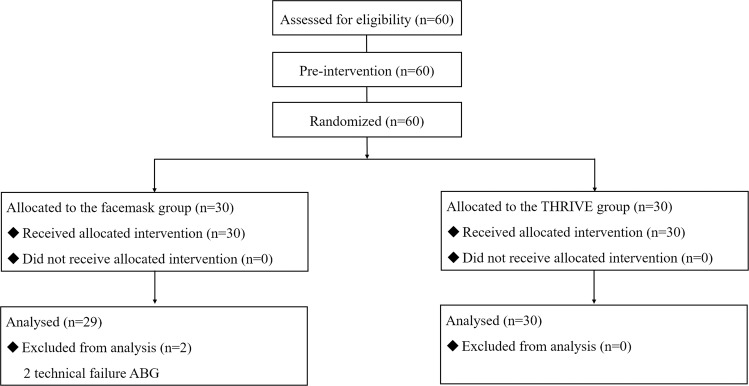
CONSORT diagram of study recruitment. THRIVE, transnasal humidified rapid insufflation ventilator exchange; ABG, arterial blood gases.

Sixty patients were included in this study. Two patients had to be excluded from the facemask group because of technical problems with the ABG monitor, leading to incorrect ABG measurements. The complete datasets included 58 patients (Fig. 1). The clinical characteristics and intubation time of the 58 patients included in the study were analysed and are summarized in Table 1.

**Table 1:** Patient Characteristics.

	THRIVE n = 30	Facemask n = 28
Age (years)	73.10 ± 5.05	70.64 ± 4.28
Sex		
Male	16 (53.3%)	17 (60.7%)
Female	14 (46.7%)	11 (39.3%)
BMI (kg m^−2^)	24.72 ± 3.03	25.03 ± 5.55
ASA status		
I	0 (0%)	1 (3.6%)
II	29 (96.7%)	27 (96.4%)
III	1 (3.3%)	0 (0%)
Modified Mallampati score		
I	22 (73.3%)	24 (85.7%)
II	8 (26.7%)	3 (10.7%)
III	0 (0%)	1 (3.6%)
Pulmonary comorbidity		
COPD	0 (0%)	0 (0%)
asthma	1 (3.3%)	1 (3.6%)
Cardiovascular comorbidity		
Hypertension	19 (63.3%)	16 (57.1%)
CHF	0 (0%)	0 (0%)
Arrhythmia	11 (51.7%)	12 (48.3%)
Breath-holding time		
<30 s	4 (13.3%)	5 (17.9%)
≥30 s	26 (86.7%)	23 (82.1%)
Hemoglobin	11.3 ± 2.0	11.7 ± 1.5
Intubation time (s)	25 (20 ~ 40)	25 (20 ~ 40)
Intubation attempts		
1	30 (100%)	28 (100%)

Data are presented as mean (SD), number (proportion) or median (inter-quartile range). Abbreviations: THRIVE, transnasal humidified rapid insufflation ventilator exchange; COPD, chronic obstructive pulmonary disease; CHF, chronic heart failure.

### Clinical outcomes

**Figure 2:**
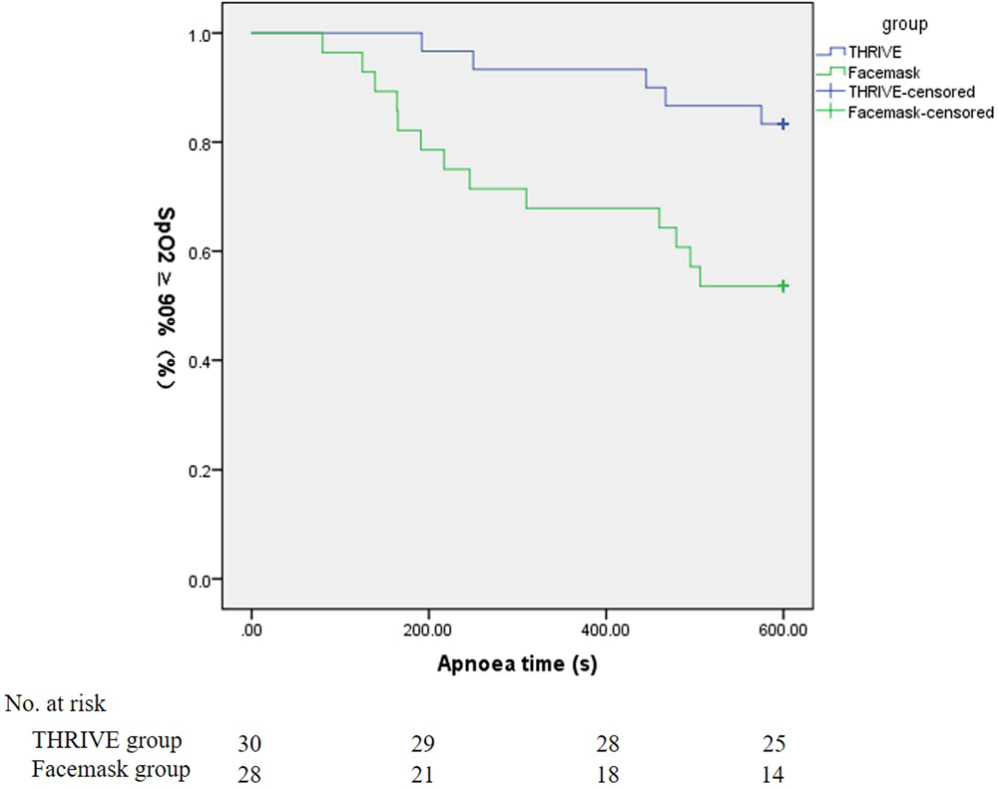
Kaplan-Meier analysis of apnoea time. THRIVE, transnasal humidified rapid insufflation ventilator exchange.

Compared with the facemask group, the THRIVE group had a significant longer apnoea time (*P* = 0.01, Fig. 2). The median apnoea times were 600 (600–600) s in the THRIVE group and 600 (231.5–600) s in the facemask group. In the THRIVE group, the duration of apnoea of 25 patients (83.8%) reached 600 s before the SpO_2_ decreased to 90%. In the facemask group, only 15 patients (53.6%) reached the termination criteria 90% SpO_2_. Compared with the facemask group, more patients in the THRIVE group reached the terminal SpO_2_ criteria (*P* = 0.014, Table 2).

**Table 2:** Termination criteria in the THRIVE group and the facemask group.

	THRIVE n = 30	Facemask n = 28	P-value
Apnea time 10 min	25 (83.3%)	15 (53.6%)	0.014*
SpO_2_ 90%	5 (16.7%)	13 (46.4%)	

Data are given as n (%). THRIVE, transnasal humidified rapid insufflation ventilator exchange. *Statistically significantly different from the facemask group, P < 0.05.

**Figure 3:**
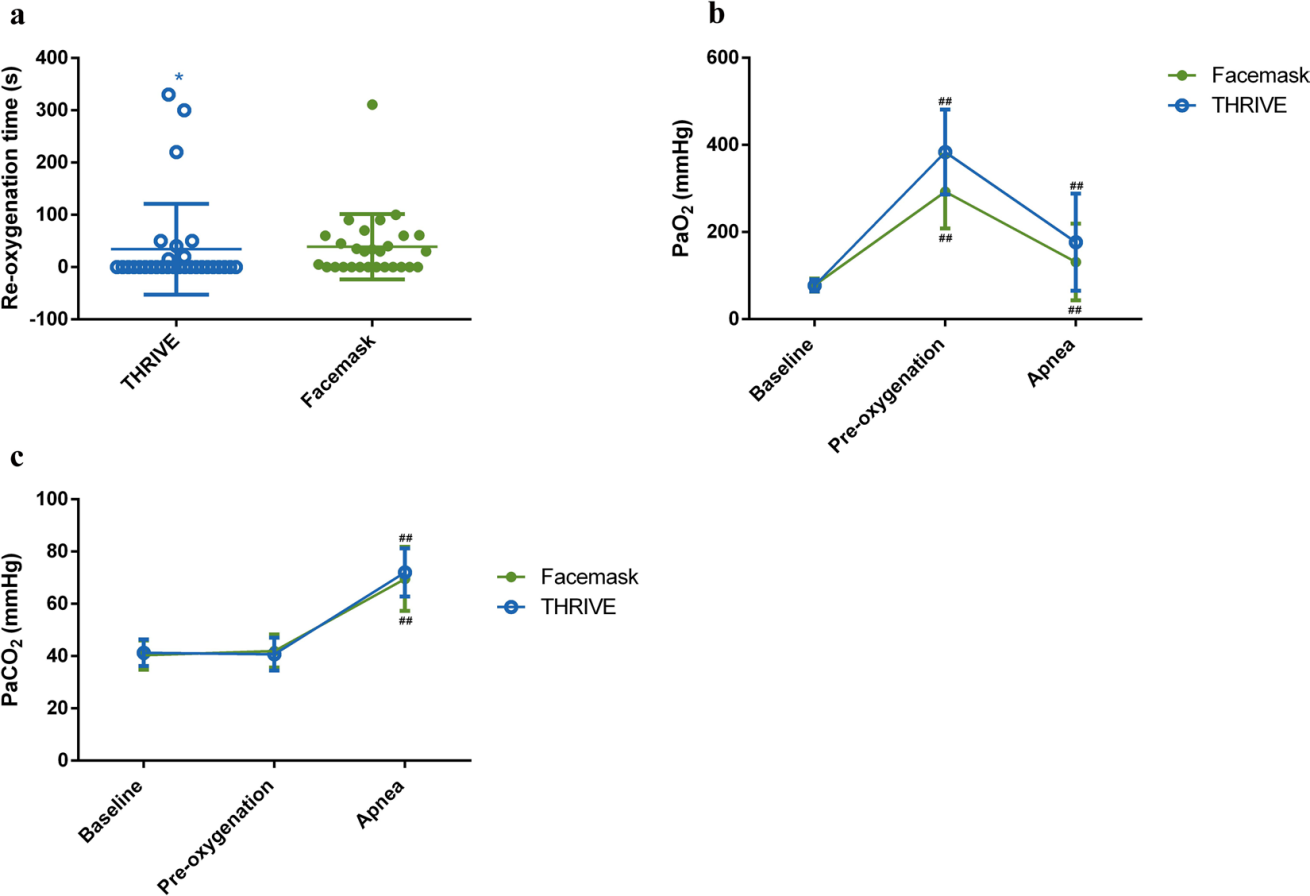
Re-oxygenation time (**a**), PaO_2_ (**b**) and PaCO_2_ (**c**) in the THRIVE and facemask groups. THRIVE, transnasal humidified rapid insufflation ventilator exchange. *Statistically significantly different from the facemask group, P < 0.05. **Statistically significantly different from the facemask group, P < 0.01^##^. Statistically significantly different from the baseline.

The median re-oxygenation time was 0 (0–16) s in the THRIVE group. In the facemask group, the median re-oxygenation time was 30 (0–60) s. The THRIVE group had a significantly shorter re-oxygenation time compared with the facemask group (*P* = 0.03, Fig. 3a).

The baseline mean (SD) PaO_2_ in the THRIVE group was 77.47 (13.80) mmHg vs. 78.18 (14.90) mmHg in the facemask group. The mean (SD) PaO_2_ was 378.37 (110.70) mmHg in the THRIVE group vs. 292.50 (84.14) mmHg in the facemask group (*P* = 0.24) after pre-oxygenation. The mean (SD) PaCO_2_ was 72.00 (9.23) mmHg in the THRIVE group vs. 69.54 (12.26) mmHg in the facemask group (*P* = 0.12) before mechanical ventilation (Fig. 3).

The mean (SD) heart rate was 66 (14) bpm in the THRIVE group vs. 66 (14) bpm in the facemask group (*P* = 0.75) after apnoea. The mean (SD) MAP was 94.8 (23.0) mmHg in the THRIVE group vs. 86.1 (17.6) mmHg in the facemask group (*P* = 0.13) after apnoea (Table 3).

**Table 3:** Heart Rate and Mean Arterial Pressure.

	THRIVE n = 30	Facemask n = 28
Heart Rate (bpm)		
Baseline	67.2 ± 8.9	69.8 ± 12.4
Pre-oxygenation	64.0 ± 9.1	66.9 ± 15.4
Apnea	65.9 ± 13.9	65.8 ± 14.4
MAP (mmHg)		
Baseline	103.1 ± 14.4	104.8 ± 11.7
Pre-oxygenation	102.1 ± 12.1	101.1 ± 12.5
Apnea	94.8 ± 23.0	86.1 ± 17.6

Data are presented as mean ± standard deviations.

Abbreviations: THRIVE, transnasal humidified rapid insufflation ventilator exchange.

### Complications

Newly presenting arrhythmias were observed in 6 of 30 patients in the THRIVE group. Among these 6 patients, single atrial premature beat appeared in 4 patients, single ventricular premature beat appeared in 1 patient, and paroxysmal supraventricular tachycardia (less than 3 s) appeared in 1 patient. Four of 28 patients developed newly presenting arrhythmia in the facemask group. Two of the four patients developed single atrial premature beat, and the other two patients developed single ventricular premature beat (*P* = 0.73, Table 4). No resistant arrhythmia was observed in either group. No nasal discomfort was reported in either the THRIVE group or the facemask group.

**Table 4:** Newly Presenting Arrhythmia and Discomfort of Nose.

	THRIVE n = 30	Facemask n = 28	*P*-value
Newly presenting arrhythmia	6 (20.0%)	4 (14.3%)	0.73
Discomfort of nose	0 (0%)	0 (0%)	

Data are given as n (%).

Abbreviations: THRIVE, transnasal humidified rapid insufflation ventilator exchange.

## Discussion

The present study showed that THRIVE significantly prolonged the apnoea time after rapid sequence anaesthesia induction in elderly patients in comparison to oxygenation via a facemask. The re-oxygenation time in the THRIVE group was also significantly lower than the re-oxygenation time in the facemask group. However, no significant differences were found on PaO_2_ and PaCO_2_ between the THRIVE group and the facemask group at the time points of pre-oxygenation and apnoea.

An increase in the duration of apnoea was observed in the THRIVE group. Improvement of the duration of apnoea might be caused by increased apnoeic oxygenation in the THRIVE group compared with the facemask group. By delivering heated and humidified oxygen with a flow rate greater than the patient’s demand for oxygen, THRIVE has been proven to provide a small amount of continuous positive airway pressure (CPAP)^20^. Continuous insufflation-induced CPAP opens the upper airways and decreases the pulmonary shunt, which is postulated to explain the mechanism underlying the increased apnoeic oxygenation provided by THRIVE^18^. To evaluate the influence of hemoglobin on the apnoea time, we examined the hemoglobin in all patients included. No significant difference on hemoglobin was found between the THRIVE group and the facemask group. A sub-analysis of apnoea time including all the 60 patients and the results were presented in the supplementary materials (S-Fig. 1 and S-Table 1). An increased duration of apnoea time was found in the THRIVE group (S-Fig. 1). In accordance with the increase of apnoea time, there were more patients with the apnoea time reaching 10 min in the THRIVE group than in the facemask group (S-Table 1). Our results further suggested that the apnoea time can be increased in elderly patients by using THRIVE when 100% oxygen was delivered via an open airway after the induction of anaesthesia. Moreover, oxygenation would be disrupted by intubation if a facemask was used to deliver oxygen^19,20^. In comparison with the facemask, THRIVE provided continuous delivery of oxygen during intubation and would be a better choice for oxygenation during intubation.

The arterial PaO_2_ after apnoea was not different between the THRIVE group and the facemask group in this study. However, it should be noted that the ABG was obtained when the SpO_2_ decreased to 90% or 10 minutes after apnoea. The effect of THRIVE on PaO_2_ may be diluted by the results from patients that SpO_2_ decreased to 90%. A sub-analysis of PaO_2_ separating patients that apnoea time reached 10 min and SpO_2_ decreased to 90% was performed. No significant difference was found on PaO_2_ in patients that apnoea time reached 10 min between the THRIVE group and the facemask group (S-Table 1). In the sub-analysis of PaO_2_ in patients that apnoea time reached 10 min, 25 patients were included in the THRIVE group and 15 patients were included in the facemask group. This result might be caused by the limited patients number included in each group. A shorter re-oxygenation time was observed in patients undergoing THRIVE compared with those in the facemask group. Compared with low-flow oxygen (10 litres min^−1^) provided by the facemask, THRIVE may achieve distal airway pressure and reduce atelectasis. The reduction in atelectasis may be the reason why the re-oxygenation time was shorter in the THRIVE group. There were 3 outliers in the THRIVE group (Fig. 3a). The re-oxygenation time in these 3 outliers were 220 s, 330 s and 300 s. The apnoea time of these 3 outliers were 467 s, 192 s and 250 s which had went out of the interquartile range of the apnoea time (600-600 s) in the THRIVE group. The outlier in the facemask group got 331 s in the re-oxygenation time and 139 s in the apnoea time which had also went out of the interquartile range of the apnoea time (231.5–600 s) in the facemask group. The result suggested that patients who had longer re-oxygenation time showed less apnoea time. No risk factors for difficult airway were found in patients with outlier values. The pulmonary function test was not performed as a routine examination in patients undergoing non thoracic surgery and without respiratory diseases related symptoms such as dyspnea, hypoxemia and cyanosis^21^. In this study, we used the breath holding time as a rough assessment test of the baseline pulmonary function. Limited to the sensitivity of breath holding test, the pulmonary dysfunction may not be accurately evaluated in some patients. We hypothesized that decreased pulmonary function might be among the reasons why these patients had longer re-oxygenation time than other patients. An analysis of SpO_2_ between the THRIVE group and the facemask group. No significant differences were found between the THRIVE group and the facemask group on SpO_2_ at the baseline, after the pre-oxygenation and after the apnoea (S-Table 3). The average SpO_2_ in both the THRIVE group and the facemask were over 90%. It should be noticed that, to avoiding complications of hypoxia and hypercapnia, the cut-off of apnoea time was set at 10 min. As the SpO_2_ had not reached 90% in the majority of patients, the re-oxygenation time was diluted by the patients that the SpO_2_ had not reached 90%. The re-oxygenation time was not a true reflection of the time needed to re-oxygenation in patients both in the THRIVE group and the facemask group. Hypercapnia can lead to hypertension, resistant arrhythmia and even cardiovascular mortality^22–24^. THRIVE has been thought to generate a cascade of vortex flows from the larger to the smaller airways, permitting gas exchange and CO_2_ elimination across the alveolo-capillary surface^11,25^. However, our study showed different results with regard to PaCO_2_, as we hypothesized that THRIVE should be superior to the facemask in terms of the elimination of CO_2_ due to the ‘washout’ effect of THRIVE on the carbon dioxide in the dead space^26^. In a study by Rudlof *et al*., the mean age of the patients was 58 years old. In our study, the average age of the patients was 72 years old. The reason why no significant difference in PaCO_2_ was found between the THRIVE group and the facemask group in our study might be linked to reduced lung compliance and increased atelectasis in the elderly.

Negative intrathoracic pressure, hypoxia and hypercapnia during apnea are risk factors for arrhythmias^24^. In comparison with the facemask group, no increased occurrence of complications, including haemodynamic instability, resistant arrhythmia or nasal discomfort, were reported in this study until the pre-set end point. These results suggested that the application of THRIVE in elderly patients was as safe as using a facemask.

This study had some limitations. First, most patients included in this study had no severe pulmonary dysfunction. The effect of THRIVE on apnoea time in elderly patients with pulmonary diseases will be evaluated in a future study. Second, no difference in the PaO_2_ was found between the THRIVE and facemask groups in our present study, which might have been the result of the limited sample size. In this study, the sample size was calculated based on our primary endpoint, which was the apnoea time. To determine the effect of THRIVE on PaO_2_, further studies with large sample sizes are still needed.

## Conclusion

During the induction of anaesthesia, THRIVE is able to extend the duration of apnoea time and provide a better pre-oxygenation effect than a facemask in the elderly without pulmonary dysfunction.

## Supplementary information

Supplementary Information.

Supplementary Information 2.
