# Supplementary material for: Transnasal humidified rapid insufflation ventilatory exchange vs. facemask oxygenation in elderly patients undergoing general anaesthesia: a randomized controlled trial
Source: Sci Rep. 2020 Apr 1;10:5745. doi: 10.1038/s41598-020-62716-2 (PMC7113239; doi:10.1038/s41598-020-62716-2)
Supplement: Supplementary file 1 — Supplementary Information. [file 41598_2020_62716_MOESM1_ESM.pdf]

Transnasal humidified rapid insufflation ventilatory exchange vs. facemask oxygenation in elderly patients undergoing general anaesthesia: a randomized controlled trial

Zhen Hua[a,*,#], Zhen Liu[a,#], Yang Li[a,b], Hongye Zhang[a], Ming Yang[a], Mingzhang Zuo, [a,*]
[a] Department of Anaesthesiology, Beijing Hospital, National Center of Gerontology, Institute of Geriatric Medicine, Chinese Academy of Medical Science, P.R. China
[b] Department of Anaesthesiology, Beijing Changping Hospital, P.R. China
[#] Zhen Hua and Zhen Liu contributed equally and share their first authorship.
[*]Corresponding author.

S-table 1 Termination criteria in the THRIVE group and the facemask group.

	THRIVE<br>n=30	Facemask<br>n=30	*P*-value
Apnea time 10 min	25 (83.3%)	15 (50%)	0.013*
SpO_2 90%	5 (16.7%)	15 (50%)	

Data are given as n (%).

Abbreviations: THRIVE, transnasal humidified rapid insufflation ventilator exchange.

**Statistically significantly different from the facemask group, P < 0.05*

S-table 2 Sub-analysis of PaO_2 on patients that apnea time reached 10 min and patients that SpO_2 decreased to 90%.

PaO_2 (mmHg)	THRIVE	Facemask	*P*-value
Apnea time 10 min*	165 (93 ~ 269)	160 (135.5 ~ 254)	0.80
SpO_2 90%#	62 (56 ~ 66)	58 (54 ~ 74)	0.57

Data are given as median (interquartile range).

Abbreviations: THRIVE, transnasal humidified rapid insufflation ventilator exchange.

* *n=25 in the THRIVE group; n=15 in the Facemask group.*

\# *n=5 in the THRIVE group; n=13 in the Facemask group.*

S-table 3. Analysis of SpO_2 in the THRIVE group and Facemask group.

SpO_2 (%)	THRIVE n=30	Facemask n=28
Baseline	95.1 ± 2.1	95.8 ± 2.5
Pre-oxygenation	99.8 ± 0.5	99.7 ± 0.5
Apnea	96.3 ± 4.1	94.0 ± 4.6

Data are presented as mean ± standard deviations.

Abbreviations: THRIVE, transnasal humidified rapid insufflation ventilator exchange
