# Supplementary figures and images for: Transnasal humidified rapid insufflation ventilatory exchange vs. facemask oxygenation in elderly patients undergoing general anaesthesia: a randomized controlled trial
Source: Sci Rep. 2020 Apr 1;10:5745. doi: 10.1038/s41598-020-62716-2 (PMC7113239; doi:10.1038/s41598-020-62716-2)

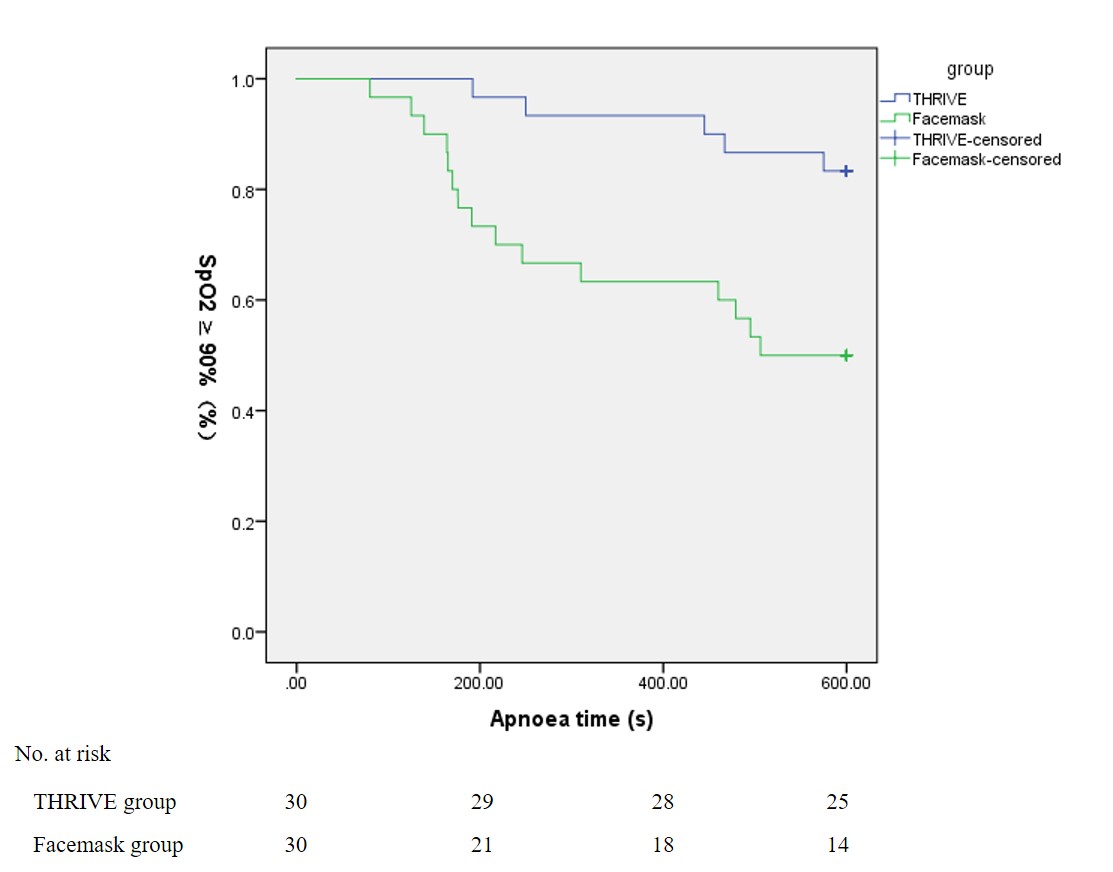

Supplement: Supplementary file 2 — Supplementary Information 2. [file 41598_2020_62716_MOESM2_ESM.jpg]
